# Injury Metrics for Assessing the Risk of Acute Subdural Hematoma in Traumatic Events

**DOI:** 10.3390/ijerph182413296

**Published:** 2021-12-17

**Authors:** Silvia García-Vilana, David Sánchez-Molina, Juan Velázquez-Ameijide, Jordi Llumà

**Affiliations:** Campus EEBE, Universitat Politècnica de Catalunya, Av. Eduard Maristany, 16, 08019 Barcelona, Spain; silvia.garcia.vilana@upc.edu (S.G.-V.); juan.velazquez@upc.edu (J.V.-A.); jordi.lluma@upc.edu (J.L.)

**Keywords:** bridging veins, TBI, injury metrics, damage metrics, biomechanics, strain rate dependent materials

## Abstract

Worldwide, the ocurrence of acute subdural hematomas (ASDHs) in road traffic crashes is a major public health problem. ASDHs are usually produced by loss of structural integrity of one of the cerebral bridging veins (CBVs) linking the parasagittal sinus to the brain. Therefore, to assess the risk of ASDH it is important to know the mechanical conditions to which the CBVs are subjected during a potentially traumatic event (such as a traffic accident or a fall from height). Recently, new studies on CBVs have been published allowing much more accurate prediction of the likelihood of mechanical failure of CBVs. These new data can be used to propose new damage metrics, which make more accurate predictions about the probability of occurrence of ASDH in road crashes. This would allow a better assessement of the effects of passive safety countermeasures and, consequently, to improve vehicle restraint systems. Currently, some widely used damage metrics are based on partially obsolete data and measurements of the mechanical behavior of CBVs that have not been confirmed by subsequent studies. This paper proposes a revision of some existing metrics and constructs a new metric based on more accurate recent data on the mechanical failure of human CBVs.

## 1. Introduction

Road traffic crashes and their sequelae are a major problem of Public Health [1]. Every year more than one million people pass away in road traffic collisions and, worldwide, about ten million people are injured or disabled by these traumatic events [2]. A large number of road traffic fatalities are associated with traumatic brain injuries (TBI). About a 33.6% of the road traffic injuries produce some type of TBI (the exact figure varies from region to region, ranging from 28.9% in Northern America to 34.4% in Africa) [3]. Globally, the total number of TBI events due to all causes affects worldwide between 64 and 74 million people suffer some form of TBI each year. One of the most common severe types of TBI is acute subdural hematoma (ASDH), caused by rupture of blood vessels connecting the sagittal sinus to the brain [4]. The incidence of ASDH in all non-missile head injuries ranges from 26% to 63% [5,6], and the mortality rate ranges from 30% to 90% [7]. These figures suggest that road crashes cause some 16 million events with subdural hematoma, 1.25 million being severe cases. Due to these figures, it can be concluded that ASDH represents an important part of the Public Health problem of road traffic crashes.

Within the automotive industry, a common approach to mitigate TBI occurrence has been the inclusion of passive and active safety systems, as well as the improvement of restraint systems, to minimize the accelerations and stress to the body in a traffic collision. Different crash tests, as well as finite element models, were used to assess the effectiveness of these countermeasures. Specifically, in the research of the effects of road collisions, the so-called finite element head models (FEHMs) have been used [8,9]. FEHMs can estimate from the acceleration data from real crash tests how much stress is induced on the various parts of the body and in particular on the anatomical structures contained within the skull. The computational results obtained from FEHM can be compared with the estimated limits of mechanical strength conducted on biological tissues. Therefore, the FEHMs allow the evaluation of *risk indexes*, also called *injury metrics*, from which the probabilities of injury or mechanical failure in different structures of the brain can be calibrated. This is the approach followed in this article. Injury metrics can improve the design of restraint systems and can provide reference values for limits in vehicle regulations [10].

For these injury metrics to make accurate predictions, more and better constitutive models are needed for the mechanical behavior of tissues. Moreover, accurate empirical data are needed to establish the physiological ranges of strength of anatomical structures located inside the head. Such data can only be obtained by direct mechanical testing on biological tissue specimens.

In this article, we will focus on some of the most widely used injury metrics to assess the probability of ASDH occurrence. Statistical analysis will lead to propose an improvement of existing metrics and even a new injury metric based on more recent data and with higher accuracy than the data available when some of the initial injury metrics were proposed. In addition, it will be shown along the paper, that there are new usable data to improve predictions about the occurrence of ASDH for traumatic events. This improvement in prediction could be used to improve vehicle regulations and restraint systems in order to have an impact on public health: regulatory changes in vehicle safety systems and improved design of restraint systems, would reduce ASDH figures associated to road traffic crashes.

## 2. Data and Methods

### 2.1. Materials and Data

For this study, previously published data of human cerebral bridging veins (CBVs) obtained by the authors themselves [11] and by other authors [12] were used.

For moderate strain rate, the data were previously obtained by the authors themselves from tensile tests of CBVs. These specimens were dissected from different sections of the meningeal-cortex space, obtained from nine autopsies of post-mortem human subjects (PMHS) conducted in the Forensic Pathology Service of the Legal Medicine and Forensic Science Institute of Catalonia For high or very high strain rate, the data published by Monea et al. [12] were used. Specifically, the data chosen were the tensile tests with strain rate in the range of $10<\dot{\varepsilon }<150$ s${}^{-1}$. This strain rate range chosen is because simulations of pedestrian collisions show that the observed range of strain rate during the traumatic event rarely exceeds 200 s${}^{-1}$).

### 2.2. Injury Metrics

An injury metric to assess TBI is a real-valued functional which depends on the linear acceleration of the skull a(t) and the rotational acceleration of the skull α(t) which is used to estimate the probability of a specific type of TBI.

In practice, two types of injury metrics are commonly used for injury prediction: *empirical metrics* and *analytical metrics*. Empirical metrics are simpler and can be computed from purely kinematic data, while analytical metrics compute an injury measure based on simulated strain or stress in tissue structures, by means of an adequate FEHM. In this article, we compare three analytical injury metrics and their predictions for a computational dataset calculated using the SIMon model, a FEHM developed by Takhounts and coworkers [13,14].

In addition, there are some mathematical properties that make a metric suitable, which we will examine for the three metrics. As a mathematical functional, the suitable properties of an injury metric are scalability, continuity and convexity [15] (see Appendix A). Scalability and continuity are intuitive, while convexity is required for the existence of an acceleration curve which minimizes the damage under specific conditions and, therefore, the value of the injury metric. Specific attention was placed on ensuring that the proposed metrics and enhancements to existing ones satisfy these suitability requirements.

The occurrence of ASDHs associated with traumatic events is clearly correlated to the mechanical failure of cerebral parasagittal bridging veins (CBVs). The mechanical failure of a blood vessel of this type generally causes a major hemorrhage in the meningeal-cortex space, resulting in an ASDH, a potentially life-threatening situation for the subject who has suffered the traumatic event.

The most widely used metric for estimating the probability of mechanical failure of CBVs has been the *relative motion damage measure* (RMDM), first introduced in 2003 [13,16]. This metric can be written as:

(1) $${\mathrm{RMDM}}_{t}=\max_{k\in N_{v}}\frac{{\langle \varepsilon_{k}\left(t\right)\rangle }_{+}}{E_{u}\left(\log_{10}{\dot{\varepsilon }}_{k}\left(t\right)\right)}$$

where $N_{v}=\left\{1,\cdots ,n\right\}$ is the set of CBVs used in the model, ${\langle \cdot \rangle }_{+}$ are the Macaulay brackets, used to define the ramp function ${\langle x\rangle }_{+}=\max\left\{0,x\right\}$, $\varepsilon_{k}\left(t\right)$ the instantaneous strain of *k*th vein, $E_{u}\left(x\right)=0.0608x^{2}-0.4414x+0.9872$ is the Löwenhielm-Takhounts failure function [13,17], and ${\dot{\varepsilon }}_{k}\left(t\right)$ the *k*th strain rate.

This metric has been extensively used in many subsequent empirical and theoretical studies which showed the usefulness of RMDM for assessing traumatic events [15,18,19,20,21]. The basic idea of this metric was to calculate the strain value of the CBVs and compare the obtained value with the value Löwenhielm–Takhounts failure function at the instantaneous strain rate. However, there is a problem with the original data, as Löwenhielm himself in a later study discarded the dependence on strain rate from their previous work due to methodological problems [22]; this fact seems to have been largely ignored in the literature using RMDM to assess ASDH risk. Moreover, Löwenhielm found that the ultimate strain was reduced from about 70% to 20% with increasing strain rate from 1 s${}^{-1}$ to 1000 s${}^{-1}$, although other much more recent studies, using better measurement techniques, show a slight increase in ultimate strain with strain rate [11,12], contradicting the original findings of Löwenhielm.

In fact, it is important to note that, after Löwenhielm’s work, a good number of published studies of the mechanical properties of CBVs have been done [11,12,23,24,25]. All the newer studies have much better precision data than the first studies, essentially due to improved measurement accuracy, so these data can be properly used to propose a more accurate and suitable injury metric.

### 2.3. Proposed Injury Metric

As mentioned before, RMDM used the idea that excessive stretching along the longitudinal direction of a CBV was the cause of its mechanical failure. Furthermore, a conjecture on how ultimate strain decreases with increasing strain rate in Löwenhielm’s empirical data [13,17] led to define the RMDM as the ratio of the instantaneous strain to the expected value of ultimate strain. Actually, the same idea can be applied to longitudinal stress and ultimate stress (or, equivalently, to current axial force and ultimate axial force). Recent data show that the ultimate stress $S_{u}$ depends on the strain rate [11,25], and a statistical analysis of the expected value of $S_{u}$ and its variation in terms of the strain rate allows to build new injury metrics on more accurate and precise recent data than the available data from Löwenhielm. Let $E(S_{u}|\dot{\varepsilon })$ be the expected value of ultimate strain $S_{u}$, given a strain rate $\dot{\varepsilon }$, then, a possible injury metric would be to divide the instantaneous stress by the expected value for ultimate stress at the current strain rate. In Section 3.1, it is shown that $S_{u}=S_{u}^{*}\cdot \phi \left(\dot{\varepsilon }\right)$ so an analysis of the intrinsic magnitude $S_{u}^{*}$ provides the adequate expected value (see Section 3.2).

The above reasoning allows us to construct a risk index for a single CBV. However, in the brain there are more than a dozen of CBVs, and to assess the possibility of a ASDH would require an analysis of the probability that at least one vessel connecting the parasagittal sinus to the brain presents a mechanical failure. In a traumatic event in which the skull and brain undergo large accelerations, the probabilities of failure of the different CBVs are not independent, because the axial forces experienced by the veins depend on the global forces produced in the interaction of the whole brain and skull. Although if we know the forces to which each individual CBV is subjected, a common situation in FEHM simulations, then it turns out that the conditional expected values $E(S_{u,i}|{\dot{\varepsilon }}_{i},F_{i})$ for the mechanical failure of each CBV with respect to its strain rate and the applied force are independent variables (since the failure of a specific CBV after having exceeded its strength limit does not affect the other CBVs). Now, let us call $p_{i}\left(F_{i},{\dot{\varepsilon }}_{i}\right)$ the probability of failure of the *i*-th CBV (estimated from empirical data in Section 3.2), knowing the values of the applied axial force and strain rate, and suppose that there are $n=|N_{v}|$ specimens of CBVs in the whole brain; then the probability $P_{0}$ of no ASDH would be equivalent to none of the CBVs exhibiting mechanical failure. Therefore, the probability of no ASDH would be the product $P_{0}=\left(1-p_{1}\right)\cdots \left(1-p_{n}\right)$ and the complementary probability ($1-P_{0}$) would be the probability of occurrence of an ASDH because any of the *n* CBVs exhibited mechanical failure. Thus, we have for the probability of occurrence of an ASDH:

(2) $$P\left(\mathrm{ADSH}|F,\dot{\varepsilon }\right)=1-\left(1-p_{1}\right)\cdots \left(1-p_{n}\right)=1-\prod_{i=1}^{n}\left(1-p_{i}\left(F_{i},{\dot{\varepsilon }}_{i}\right)\right)$$

where $F=(F_{1},\cdots ,F_{n})$ is the array of axial forces and $\dot{\varepsilon }=\left({\dot{\varepsilon }}_{1},\cdots ,{\dot{\varepsilon }}_{n}\right)$ is the set of strain rates of the CBVs. If we designate the Cumulative Distribution Function (CDF) for the strength $\Phi_{F_{u}^{*}}$, estimated from empirical data (see Section 3.2), the value of the strength that equals the probability in (2) will be the new risk measure we call *Bridging Vein Damage Metric* (BVDM):

(3) $$\mathrm{BVDM}=\Phi_{F_{u}^{*}}^{-1}\left(P\left(\mathrm{ADSH}|F,\dot{\varepsilon }\right)\right)$$

It is interesting that the specific empirical form of the distribution $\Phi_{F_{u}^{*}}$ leads to a closed, logical and very manageable formula for BVDM, see Equation (9).

With resecto to the estimated risk, it is important to note that some studies using RMDM simply take the risk of ASDH as the highest value of this injury metrics, but that underestimates the risk. If we go back to the Equation (2), it can be easily shown by induction that for $0<p_{i}<1$:

(4) $$\left(1-p_{1}\right)\cdots \left(1-p_{n}\right)\geq \max\left\{p_{1},\cdots ,p_{n}\right\}$$

In fact, only when one of the probabilities is much larger than the others are, then the following approximation is possible:

(5) $$\left(1-p_{1}\right)\cdots \left(1-p_{n}\right)\approx \max\left\{p_{1},\cdots ,p_{n}\right\}=p_{\max}$$

but when all the CBVs have similar and relatively high risks $P\left(\mathrm{ADSH}|F,\dot{\varepsilon }\right)>>p_{\max}$. i.e., the overall risk of ASDH can be much higher than the risk estimated only from the bridging vein in the most critical situation. This allows us to give an improved version of RMDM, the RMDM${}_{eq}^{*}$, calculated as:

(6) $${\mathrm{RMDM}}_{eq}^{*}=\phi^{-1}(1-\left(1-\phi \left({\mathrm{RMDM}}_{1}\right)\right)\cdots \left(1-\phi \left({\mathrm{RMDM}}_{n}\right)\right)$$

where ϕ(RMDM) is the probability of failure for a CBV from the RMDM risk curve. The risk curve was estimated with empirical data in [13]. From the same risk curve, we estimate the probabilities ${\hat{p}}_{i}=\phi \left({\mathrm{RMDM}}_{i}\right)$ of independent failure of each CBV from its specific RMDM${}_{i}$ value.

## 3. Results

This section details how to separate the effect of intrinsic factors from effect of the strain rate $\dot{\varepsilon }$ on the ultimate stress $S_{u}$ of a CBV specimen (Section 3.1). This separation allowed us to find the intrinsic ultimate stress, on which the damage metric is built. For this purpose, the probabilities of finding an ASDH due to mechanical failure of some CBV are calculated (Section 3.2). Finally, a comparison between the predictions of RMDM and BVDM is presented in Section 3.3.

### 3.1. Dependence of Ultimate Stress on Strain Rate

**Figure 1:**
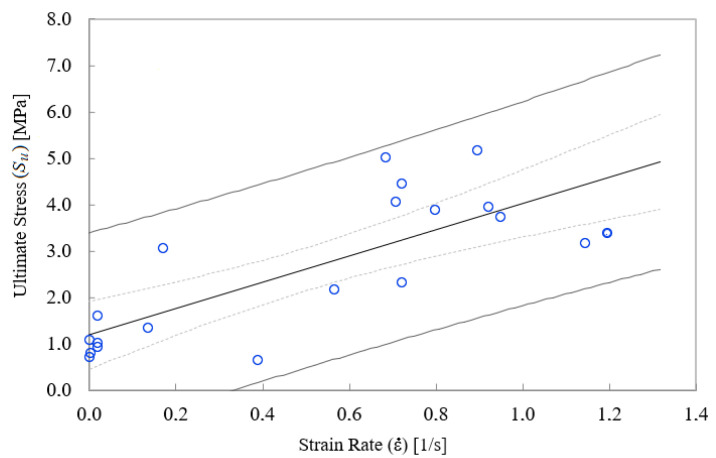
Scatterplot of ultimate stress vs. strain rate for low values of strain-rate a significant relation exists (*p*-value < 0.001).

**Figure 2:**
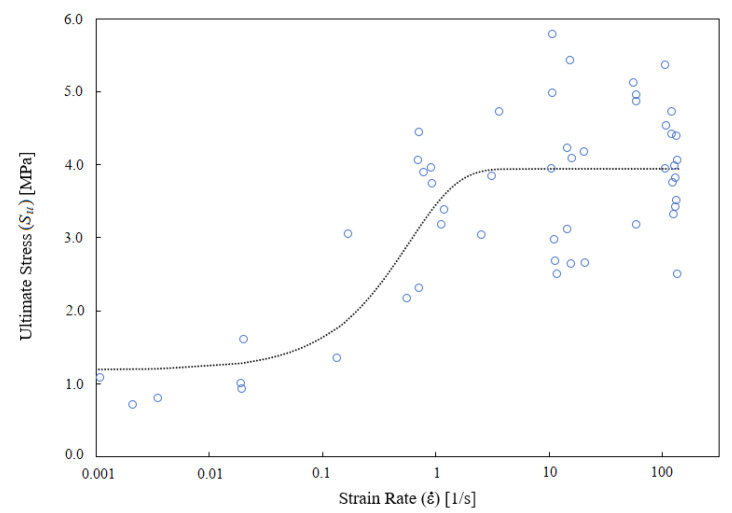
The nonlinear relation of ultimate stress and strain rate, for higher values of strain-rate $\dot{\varepsilon }>10$ s${}^{-1}$ the ultimate stress seems independent of the strain rate. The SRDF $\varphi (\dot{\varepsilon })$ is proportional to the black dotted line.

For low strain rate, the analysis of empirical data for ultimate stress $S_{u}$ shows a significant increasing trend (*p* < 0.001) of this magnitude with strain rate (see Figure 1). The data from Monea et al. also show such an increasing trend [12], although in the range studied $50<\dot{\varepsilon }<200$ s${}^{-1}$ the effect of strain rate does not reach to be significant. This suggests that there is a significant increase in $S_{u}$ for low strain rate, but that effect is “saturated” for values of the strain rate $\dot{\varepsilon }>10-25$ s${}^{-1}$, as shows Figure 2.

For this reason, a *strain-rate dependence function* (SRDF) $\varphi (\dot{\varepsilon })$ is constructed, which interpolates the high sensitivity of $S_{u}$ at low strain rate, with the relative insensitivity of the same magnitude at high strain rate. In particular, the following factorization is used:

(7) $$S_{u}=S_{u}^{*}\cdot \varphi \left(\dot{\varepsilon }\right)=S_{u}^{*}\;\left[\alpha_{0}-\left(1-\alpha_{0}\right)\left(1-e^{-\gamma \dot{\varepsilon }}\right)\right]$$

The first factor $S_{u}^{*}$ is called the intrinsic ultimate stress and the second one, in square brackets, is the SRDF $\varphi (\dot{\varepsilon })$, which captures the average effect of the strain rate (the fitted values for the parameters are $\alpha_{0}=0.256$ and γ=1.721). Thus, it is proposed that the maximum stress supported by a specimen of CBV is a combination of two types of factors: the intrinsic factors of the specimen quantified by $S_{u}^{*}$, and the effect of the strain rate that is accounted for by the SRDF given by Equation (7). Note that the same equation implies that $\alpha_{0}<\varphi \left(\dot{\varepsilon }\right)<1.$ Furthermore, the factorization proposed in Equation (7) allows to statistically analyze the intrinsic mechanical failure of each CBV specimen by looking for the distribution function of the $S_{u}^{*}$ variable.

### 3.2. Distribution of the Intrinsic Ultimate Stress

It is a well-known fact that when examining a population of the same type of force-resistant elements possessing a certain micro-structure, the ultimate stress is often distributed according to a generalized extreme value distribution (GEV distribution). Interestingly, the fundamental theorem of *extreme value theory* (EVT), the Fisher–Tippett–Gnedenko theorem [26], has its origin in observations of ultimate strength, since the British statistician L. H. C. Tippett working for the British Cotton Industry Research Association developed some of the basic ideas of EVT when he dealt with the problem of textile fiber strength [27,28]. Similarly, EVT considerations seem to explain why in many practical situations the ultimate stress of many ceramic materials is given by a Weibull distribution [29,30,31], which is a particular case of GEV. Even in collagenous fabrics, there are theoretical reasons to expect the ultimate stress to conform to a GEV distributiib [32].

**Figure 3:**
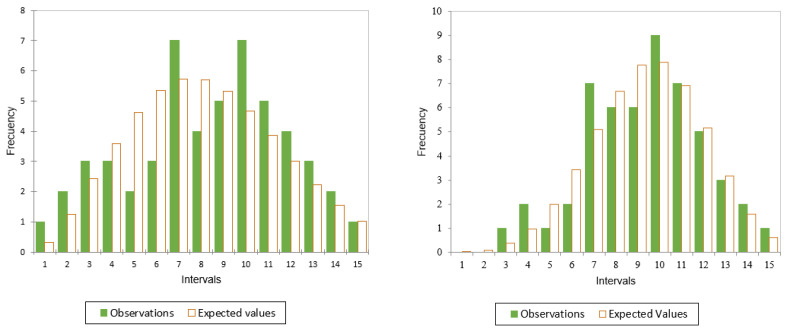
Comparison of the distributions for the ultimate axial force ($F_{u}$) and the intrinsic ultimate force ($F_{u}^{*}$).

A total sample of N=52 values of pairs $\left(\dot{\varepsilon },F_{u}\right)$ were used for the statistical analysis. From the known axial force values, the probability distribution functions were sought for the values of the ultimate axial force ($F_{u}=S_{u}\cdot A$, being *A* the cross-section area of the CBV and the intrinsic ultimate axial force $F_{u}^{*}=S_{u}^{*}\cdot A=F_{u}/\phi \left(\dot{\varepsilon }\right)$, see Equation (7). Figure 3 shows the distributions for both variables: $F_{u}$ and $F_{u}^{*}$. The larger variance found for $F_{u}$ is due to the fact that the effect of the strain rate is not being taken into account. When $F_{u}^{*}$ is considered instead of $F_{u}$, the coefficient of variation (CV = standard deviation/average) is reduced from CV = 0.450 (for $F_{u}$) to CV = 0.293 (for $F_{u}^{*}$). The larger variance in $F_{u}$ is due to the variation associated with different values of strain rate.

**Figure 4:**
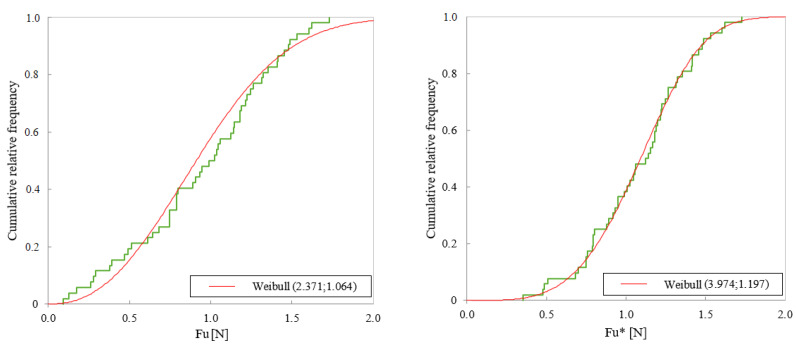
Comparison of the Cumulative Distribution Function (CDF) for the ultimate axial force ($F_{u}$) and the intrinsic ultimate force ($F_{u}^{*}$), red line: Weibull distribution, green line: empirical distribution function.

From all the distributions which were examined, the distribution that better represents the data was a Weibull distribution, confirming the expectations mentioned at the beginning of this section. Thus, the CDF for the intrinsic ultimate axial force is $F_{u}^{*}$:

(8) $$\Phi_{F_{u}^{*}}\left(F\right)=P\left(F_{u}^{*}\leq F\right)=1-e^{-{\left(F/F_{0}\right)}^{p}}$$

where the fitted values for the parameters are: $F_{0}=1.197$ [N] for the scale parameter and p=3.974 for the shape parameter. The *p*-values obtained in the goodness-of-fit tests to a Weibull distribution were high, corroborating that the Weibull distribution can adequately represent the data: for the Kolmogorov–Smirnov test the *p*-value was 0.962 and for the chi-squared test ($\chi^{2}$) the *p*-value was 0.958. Figure 4 shows the form of the CDFs for $F_{u}$ and for $F_{u}^{*}$. The CDF for $F_{u}^{*}$ coincides with Equation (8).

Once the probability of mechanical failure for an individual CBV is known, the problem of finding an injury metric that considers the possible damage in *n* independent CBVs is easy. The problem was discussed in Section 2.3, where the Equation (3) was proposed. Interestingly, the fact that CDF for the intrinsic force $\Phi_{F_{u}^{*}}$ is a Weibull distribution implies that the BVDM is given in a natural way by an *p*-norm of the intrinsic axial forces:

(9) $$\mathrm{BVDM}=\max_{t\in R}{∥F^{*}\left(t\right)∥}_{p}=\max_{t\in R}{\left(|F_{1}^{*}{|}^{p}+\cdots +{\left|F_{n}^{*}\right|}^{p}\right)}^{1/p}$$

This formula is proved in Appendix A. In the Formula (9), the intrinsic axial forces are given by the ratio of the nominal axial force by the SRDF, i.e., $F_{i}^{*}=F_{i}/\varphi \left({\dot{\varepsilon }}_{i}\right)$, so the above equation can be written in terms of ordinary axial forces as:

(10) $$\mathrm{BVDM}=\max_{t\in R}{\left(\sum_{i=1}^{n}{\left|\frac{F_{i}\left(t\right)}{\alpha_{0}-\left(1-\alpha_{0}\right)\left(1-e^{-\gamma {\dot{\varepsilon }}_{i}}\right)}\right|}^{p}\right)}^{1/p}$$

It is clear that BVDM is a scalable and continuous function. Moreover, in the Appendix A it is justified that, for p≥1, it is also a convex function, under some reasonable assumptions. All these properties make BVDM a mathematically suitable metric in the sense of [15]. As for the suitability of RMDM, it was previously discussed in the same reference.

### 3.3. Comparison of the Metrics in a Fall from a Height

**Figure 5:**
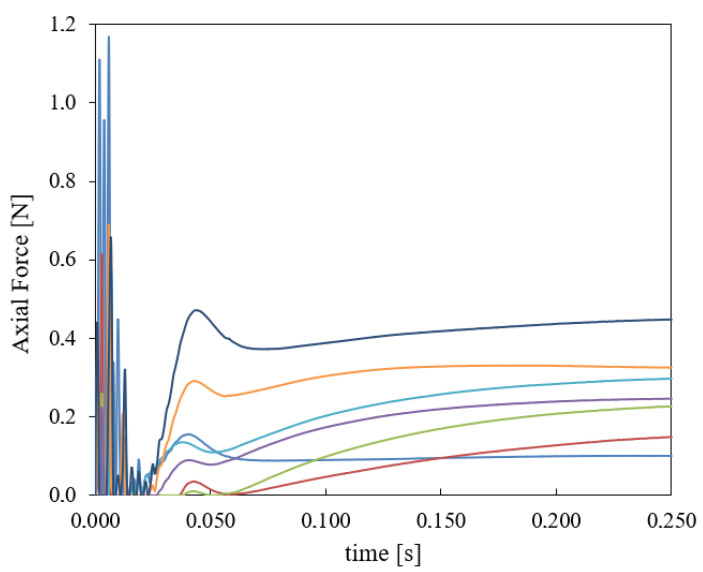
Axial Forces on the CBVs of the SIMon model (FEHM), computed for the described fall from a height of 2.5 m against a ground of ballast stiffness $k_{b}=40\;{N/\mathrm{cm}}^{3}$. One line is presented for each pair of CBVs of the FEHM. Each color line represents a different CBV, the forces differ because the CBVs are located in different parts of the brain.

In order to compare the above metrics (the conventional RMDM, the equivalent global RMDM${}_{eq}^{*}$ of Section 2.3, and the proposed new metric BVDM), some simulations were conducted. We considered the fall of a person from a certain height, being hit on the head first and, as a consequence, being stopped by the ground. More specifically, we used the SIMon FEHM [14] to simulate the fall of a person of 1.80 m falling from a height of 2.50 m. The center of gravity of the person follows a parabolic trajectory, while the body is rotating at ω=2.86 s${}^{-1}$. The ground is tough and has a ballast stiffness of $k_{b}=40$ N/cm${}^{3}$. The vertical component of the impact velocity of the head is $V_{y}=6.495$ m/s and the horizontal component is $V_{x}=0.214$ m/s. The kinematic data were incorporated into the SIMon FEHM which calculated strain, stress and axial forces in the different anatomic parts of the head. The axial force curves computed by the FEHM for each of the n=14 CBVs of the SIMon model are shown in Figure 5.

**Figure 6:**
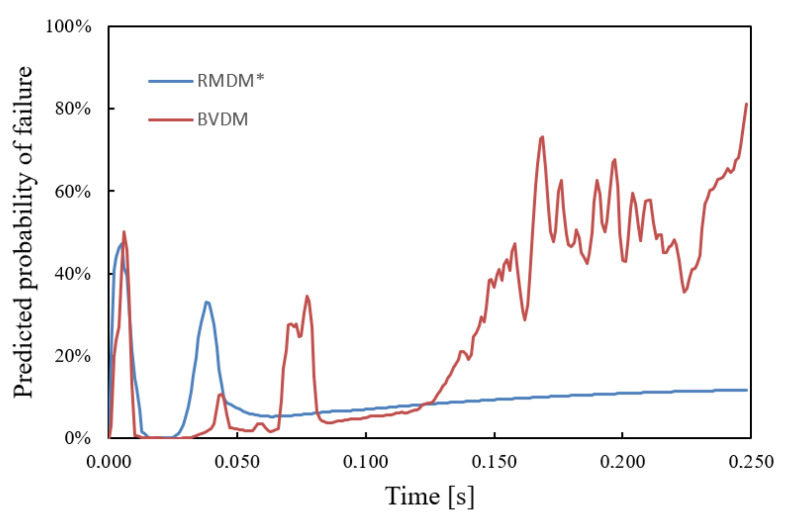
Comparison of the predictions of RMDM${}_{eq}^{*}$ and the BVDM, at the beginning of the traumatic event both metrics make similar predictions, but towards the end, when the head rebounds and the axial forces stabilize, the predictions differ considerably.

From the axial force and strain rate values, the instantaneous values of RMDM${}_{t}$, RMDM${}_{eq,t}^{*}$ and BVDM${}_{t}$ were computed. Figure 6 shows the evolution over time of the probabilities predicted by the last two metrics (RMDM is qualitatively similar to RMDM${}_{eq}^{*}$, although smaller in magnitude, because of this, its predicted probability is not represented). The maximum probability over time is obtained for the maximum of the instantaneous values of RMDM${}_{t}$ and BVDM${}_{t}$. The maximum probability for each injury metric, in Figure 6, is the estimated probability of the occurrence of an ASDH during head-on-ground impact.

From Figure 5, we see that initially, when the head hits the ground, the largest forces are registered, and the elastic compresive waves shake the brain mass inside the skull walls. Regarding the comparison between RMDM and BVDM, in the initial phase, up to 0.25 ms (see Figure 5), the RMDM${}_{eq}^{*}$ and BVDM predictions run almost parallel and the maximum ASDH probabilities reach 47.5% and 50.0%. During the next phase, where the head rebounds from the ground, the brain mass is compressed and the CBVs together with the skull stretch it. In this phase the force of the most requested CBV is 40% of the value in the initial phase, and the strain rate is considerably lower, and the RMDM and the BVDM differ markedly, since they treat the strain rate effect in a different way: the Löwenhielm-Takhounts failure function, inside Equation (1) and the SRDF inside Equation (7) differ considerably. Due to this, the risk predicted by RMDM, when the strain rate is low for t>0.100 s, is considerably smaller than the risk predicted by BVDM.

## 4. Discussion

The use of injury metrics to assess the likelihood of injury has been a growing trend over the past few decades [33,34,35,36]. In fact, a large number of injury metrics has been proposed: for diffuse axonal damage [37,38], for concussion [39], for rib breakage and thorax deformation [40,41], and even for whiplash or vertebral fracture [42]. However, the mechanical failure of CBVs has been comparatively less studied. The only widely used injury metric or risk index to assess the likelihood of ASDH is the RMDM [13,14,15]. However, the empirical data from Löwenhielm which were used to construct RMDM [13,17] are in question, as it appears that many authors have not found ultimate strain to decay noticeably with strain rate in the range $10<\dot{\varepsilon }<150\;s^{-1}$ [11,12,22,25]. Using recent data, it has been found that at low strain rate $\dot{\varepsilon }<2\;s^{-1}$, $S_{u}$, increases significantly [11] and from $\dot{\varepsilon }>10$ s${}^{-1}$ no significant changes are detected [12]. This agrees with the case of other tissues, where the ultimate strength also increases with strain rate [43].

For our analysis, an estimation of the CDF for both the ultimate stress $S_{u}$ and the ultimate axial force $F_{u}$ was made from the empirical data of recent studies of CBVs. Moreover, the same data allowed us to distinguish the effect of intrinsic factors, independent of the strain rate and affecting the failure of the CBVs, from the average effect of the strain rate itself. Because of this, it was easy to find a CDF for the so-called intrinsic axial force $F_{u}^{*}$. In fact, this latter CDF had a lower dispersion and presents a better fit to an expected distribution for mechanical failure (a GEV distribution). This suggests that factoring the strain rate effect is on the right track, as it allowed us to better pinpoint the mechanical failure probabilities of the CBVs. For our analysis, we preferred to use the ultimate axial force $F_{u}$ since the cross-sectional area of the CBVs also varies markedly and seemingly randomly between individuals, so the $S_{u}$ data present a variability practically as large as that of $F_{u}$. That is why in this paper we chose to work with force measures rather than stress measures, as they are more direct, simple to obtain and directly comparable to mechanical test data. It also turns out that many FEHMs use reduced models for CBVs and only compute the value of the axial force *F*. For all these reasons, the proposed new metric BVDM (9) has been formulated in terms of axial forces. However, with trivial modifications the approach followed here would be applicable to empirical data based on ultimate stress and thus, does not detract from the generality of the paper’s approach. In recent years, FEHM modeling the CBV complex in a more realistic way have appeared [8,9,44,45], and in these FEHMs the above approach can be applied just replacing ultimate axial forces with ultimate stress.

Another interesting point of our analysis is the distinction between local risk (failure in a specific CBV) and global risk (failure in any of the existing CBVs). Since any of the *n* CBVs within the brain surface could lead to an ASDH, the individual probabilities of failure of each CBV must be combined to assess the global risk of ASDH. In fact, in many cases it is insufficient to consider the risk of ASDH as the risk of the most critical CBV (especially if several CBVs have a moderate and relatively similar level of risk). For the case of the ultimate intrinsic axial force, this probabilistic analysis leads to an injury metrics that is a scalable, continuous and convex function of the forces, that is particularly simple (*p*-norm in the space of ultimate axial forces), as the experimental failure data seem to be given by a Weibull distribution.

This combination of individual risks in the overall aggregated risk is important, since, in addition to SIMon FEHM which includes 7 element pairs for the CBVs, most recognized FEHMs use multiple elements to model the CBVs. In this sense, the KTH FEHM (S. Kleiven) [46] uses 11 pairs of CBVs, the UCDBTM (University College Dublin) [47,48] also uses 11 pairs, the WSUBIM (Wayne State University) [49] uses 8 pairs, and the YEAHM (University of Aveiro) uses 9 pairs [9,50]. This last FEHM substantially improves both the geometrical and mechanical realism of the CBVs.

In addition, a comparison of the overall risk calculated using RMDM${}_{\max}$, RMDM${}_{eq}^{*}$ and the proposed new injury metric, BVDM, has been presented. This comparison estimates the risks for a fall from height where the head strikes a floor of known stiffness, using the SIMon FEHM to calculate internally induced forces for CBVs. The comparison showed that ASDH probabilities estimated from RMDM and a reduction in ultimate strain can differ markedly from predictions based on more recent data based on ultimate stress or ultimate axial force.

A limitation of the empirical data used is that viscoelastic effects in CBVs have been insufficiently studied [25]. Moreover, failure data available correspond to loading curves in which the strain rate is approximately constant. However, as shown in Figure 5 at the beginning of a strong head impact highly oscillating values of the force are observed, partly because the existing FEHMs do not model any viscoelastic effect in CBVs. That could be producing somewhat larger instantaneous force peaks than the actually existent ones. It should also be investigated whether the deduced failure force for monotonic loading curves needs some correction because of the viscoelastic preconditioning also discussed in [25,51].

For these reasons, further work is needed to corroborate whether in mechanical tests with PMHS the BVDM that frequently predicts a higher risk than the RMDM for low strain rate could outperform the metrics currently in use. At the present stage and based on experiments on isolated CBVs, it is not completely sure to say that BVDM will perform better than RMDM, even if it is based on recent measurements and with lower measurement errors.

## 5. Conclusions

The number of ASDH occurrences due to road traffic crashes constitute an important Public Health issue. Although the usual approach of automotive industry based on injury metrics seems well founded from a theoretical point of view, it should make use of most recent data on the mechanical properties of CBVs, because the measurements in more modern tests are more accurate. Specifically, the injury metric most commonly used to predict ASDH is based on old data that subsequent research does not seem to have validate. Therefore, FEHMs are very helpful to figure out whether a metric based on more modern and accurate constitutive model data is better than the old ones. Here, the BVDM has been proposed to make more accurate predictions, and providing an additional guide for the design of restraint systems.

This paper explains how to use modern data to find intrinsic forces and stress, independent of strain rate, which can be used to construct a new injury metric that satisfies the reasonable mathematical requirements for any injury metric. All these improvements could play a role in vehicle regulations that would eventually reduce the percentage of more serious TBI cases in which ASDH appears as a consequence of road traffic crashes.

## Data Availability

The data are available in the UPC repository https://upcommons.upc.edu, accessed on 5 November 2021, search under the name of the article.

## Abbreviations

The following abbreviations have been used in the manuscript:

ASDH	Acute Subdural Hematoma
BVDM	Bridging Vein Damage Metric
CBV(s)	Cerebral Bridging Vein(s)
CDF(s)	Cumulative Distribution Function(s)
EVT	Extreme Value Theory
FEHM	Finite Element Head Model
GEV	Generalized Extreme Value
PMHS	Post-Mortem Human Subject
RMDM	Relative Motion Damage Measure
SRDF	Strain Rate Dependence Function
TBI	Traumatic Brain Injury

## Appendix A. Suitability of BVDM

In this appendix, we show how the Formula (3) leads to Formula (9), when the variables involved are distributed according to a Weibull distribution. Moreover, we justify here that the metric associated with Equation (9), the BVDM, is a scalable, continuous and convex functional.

With respect to deduction of Formula (9), note that the CDF of the intrinsic axial force $F_{u}^{*}$ has the form:

(A1) $$p_{i}=\Phi_{F_{u}^{*}}\left(F_{i}\right)=1-e^{-{\left(F_{i}/F_{0}\right)}^{p}}$$

substituting this formula in the initial product of the Equation (4), it follows that:

(A2) $$\left(1-p_{1}\right)\cdots \left(1-p_{n}\right)=e^{-{\left(F_{1}/F_{0}\right)}^{p}}\cdots e^{-{\left(F_{n}/F_{0}\right)}^{p}}=e^{-\left[{\left(F_{1}/F_{0}\right)}^{p}+\cdots +{\left(F_{n}/F_{0}\right)}^{p}\right]}$$

and since the inverse of the function of (A1) is given by:

(A3) $$\Phi_{F_{u}^{*}}^{-1}\left(q\right)=F_{0}{\left[-\ln\left(1-q\right)\right]}^{1/p}$$

We have that, introducing the final part of the Equation (A2) in Equation (A3), we get that:

$$\begin{array}{cc} \Phi_{F_{u}^{*}}^{-1}\left(1-e^{-\left[{\left(F_{1}/F_{0}\right)}^{p}+\cdots +{\left(F_{n}/F_{0}\right)}^{p}\right]}\right) & =F_{0}{\left[{\left(\frac{F_{1}}{F_{0}}\right)}^{p}+\cdots +{\left(\frac{F_{n}}{F_{0}}\right)}^{p}\right]}^{1/p}\\ & =\frac{F_{0}}{|F_{0}|}{\left[F_{1}^{p}+\cdots +F_{n}^{p}\right]}^{1/p} \end{array}$$

This is precisely the basic form contained in Equation (9).

As for the suitability of injury metrics (9), we justify that this metric is scalable, continuous and convex. For this purpose, we make the natural assumption that the stress tensor of the CBVs (σ depends continuously on the accelerations imposed on the skull (a(t),α(t)), since the stress is obtained from an equation in partial derivatives where the skull accelerations appear in the form:

(A4) $$\mathrm{div}\left(\sigma \right)+b=\rho \frac{\partial v}{\partial t}=\rho a$$

In addition, we note that, when the accelerations are rescaled by λ>1, i.e., by imposing (λa(t),λα(t)) on the skull, the volume forces change as follows $b\mapsto b_{\lambda }=b_{0}+b_{1}\lambda +b_{2}\lambda^{2}$ [15], where $b_{0}$ represents all the terms independent of acceleration (basically weight), $b_{1}$ depends on the linear acceleration of the center of mass (the Euler and the Coriolis accelerations), and $b_{2}$ depends on the centripetal acceleration. It should also be noted that the forces in the skull are continuous functions of linear and angular accelerations, i.e., $F_{i}=f_{i}\left(a,\alpha \right)$.

For points on the periphery of the brain, we should expect those terms to have the same sign and thus always increase stress in each and every CBV. Convexity is more difficult to justify without additional assumptions. For example, wa admit that the axial force on each of the CBVs is itself a convex functional of the imposed accelerations (this is difficult to prove explicitly, given the complexity of FEHMs, but seems consistent with the observed facts). Assuming this property, it is immediate to show that the BVDM leads to a convex metric, since the *p*-norm $\left(\right|x_{1}{|}^{p}+\cdots +|x_{n}{{|}^{p})}^{1/p}$ is always a convex function for p≥1, indeed as a vector norm over $R^{n}$ it is continuous and convex:

$${∥\left(1-\lambda \right)x+\lambda y∥}_{p}\leq {\left(1-\lambda \right)∥x∥}_{p}+\lambda {∥y∥}_{p}$$

for 0<λ<1 and $x,y\in R^{n}$. Trivially, this function is also scalable, since ${∥\lambda x∥}_{p}={|\lambda |∥x∥}_{p}>{∥x∥}_{p}$, for λ>1.
